# Local Alignment Tool Based on Hadoop Framework and GPU Architecture

**DOI:** 10.1155/2014/541490

**Published:** 2014-05-14

**Authors:** Che-Lun Hung, Guan-Jie Hua

**Affiliations:** ^1^Department of Computer Science and Communication Engineering, Providence University, No. 200, Section 7, Taiwan Boulevard, Shalu District, Taichung 43301, Taiwan; ^2^Department of Computer Science and Information Engineering, Providence University, No. 200, Section 7, Taiwan Boulevard, Shalu District, Taichung 43301, Taiwan

## Abstract

With the rapid growth of next generation sequencing technologies, such as Slex, more and more data have been discovered and published. To analyze such huge data the computational performance is an important issue. Recently, many tools, such as SOAP, have been implemented on Hadoop and GPU parallel computing architectures. BLASTP is an important tool, implemented on GPU architectures, for biologists to compare protein sequences. To deal with the big biology data, it is hard to rely on single GPU. Therefore, we implement a distributed BLASTP by combining Hadoop and multi-GPUs. The experimental results present that the proposed method can improve the performance of BLASTP on single GPU, and also it can achieve high availability and fault tolerance.

## 1. Introduction

In the past decade, the sequencing technologies have been improved dramatically. An entirely new technology was developed, next generation sequencing (NGS), a fundamentally different approach to sequencing DNA and RNA much more cheaply and quickly than traditional Sanger sequencing. Meanwhile, NGS is well known as a high-throughput sequencing technology. The number of output data produced by NGS data has increased more than double each year since it was invented. In 2007, a single sequencing run could produce around one gigabase (Gb) of sequence data. By 2011, it approaches a terabase (Tb) of data produced in a single sequencing run—nearly a 1000× increase in four years. With the ability to rapidly generate large amount of sequencing data, NGS has enabled the researches in the field of biology and other closely related fields can be done at a large-scale level and also can move quickly from an idea to full data sets in a matter of hours or days [1]. As NGS becomes key player in modern biological research, the analysis of the vast amount of produced data is not an easy task and a great challenge in the field of bioinformatics. Therefore, efficient tools to cope with these big data to provide the knowledge easier and faster are essential.

With the rapid development of multicore hardware, graphics processing units (GPUs) are being used in numerous applications to enhance computational performance. GPUs have a low design cost and the increased programmability of GPUs allows them to be more flexible than FPGAs. General-purpose graphics processing units (GPGPU) programming has been successfully used in scientific computing domains, which involve a high level of numeric computation. The greatest benefit of GPUs is that the number of processing units is immense compared to those of CPUs (CPU, approximately 2–16; GPU, approximately 128–512). In 2006, Nvidia proposed the compute unified device architecture (CUDA). CUDA uses a new computing architecture named single instruction multiple threads (SIMT). This architecture allows threads to execute independent and divergent instruction streams, thus facilitating decision-based execution, which is not provided by the more common single instruction multiple data (SIMD). Many well-known tools have been reimplemented based on GPU architecture [2–4]. One of the wild-use alignment tools, BLASTP, is a heuristic algorithm to produce a local alignment for protein. BLASTP has three implementations on GPU, GPU-NCBI-BLASTP [5], CUDA-BLASTP [6], and GPU-BLASTP [7]. All three implementations achieve 4x~40x speedup over a single-thread CPU implementation of NCBI-BLAST.

Meanwhile, the software architectures of distribution computing have been developed rapidly as well. The cloud computing as a new distribution computing service concept has become popular for providing services with availability, reliability, and on-demand computation to users. The cloud computing environment can be a distributed system that has massively scalable IT-related capabilities, providing multiple external customers many services on Internet. In addition, cloud computing can be used to copy with big data and maintain high availability and fault tolerance. Hadoop [8] is one of the commonly used open source software frameworks intended to support data-intensive distributed applications. Hadoop adopts Map/Reduce programming model to process petabytes of data with thousands of nodes. Map/Reduce programming model is useful to develop parallel computing applications on cloud computing environment. In Map/Reduce model, mappers and reducers complete a task. Each mapper performs a map operation and each map operation is independent of the others. A task is split into many subtasks, and each mapper processes its subtask. Similarly, a set of reducers can perform a set of reduce operations. Reducers deal with the data produced by mappers. An important benefit of using Hadoop to develop the application is fault tolerance. Hadoop can guide jobs toward a successful completion even when individual nodes experience failure in computation. In these situations, Hadoop platform is considered as a much better solution for these real-world applications. Currently, Hadoop has been applied in various domains in bioinformatics [9–13]. Cloud-PLBS [14] is a cloud service that combines the SMAP [15–17] and Hadoop frameworks for 3D ligand binding site comparison and similarity searching of a structural proteome. This platform is computationally more efficient than standard SMAP. Hung and Lin [12] proposed a parallel protein structure alignment service based on the Hadoop distribution framework. This service includes a protein structure alignment algorithm, a refinement algorithm, and a Map/Reduce programming model. The computational performance of their service is proportional to the number of processors used in their cloud platform.

In this paper, we combine these two different heterogeneous architectures, software architecture-Hadoop framework and hardware architecture-GPU, to develop a high performance cloud computing service for protein sequence alignment. In this cloud service, each mapper performs BLASTP and a reducer collects all resulting alignments produced by mappers. The mappers work simultaneously. By using Hadoop, the proposed GPU based bioinformatics cloud service can recover the comparison job from a crashed GPU host by assigning this job to other health GPU hosts. This cloud platform can achieve high performance, scalability, and availability. The experimental results present that the computational performance of the proposed service can be enhanced by using Hadoop and GPU architecture.

## 2. Method

In the work, we integrate BLASTP with Hadoop. Hadoop framework works with mappers and reducer. Mappers perform BLASTP on GPU, and reducer collects all alignment results produced by mappers. Despite Hadoop distribution computing framework, performance of BLASTP can be enhanced by multiple mappers. Hadoop guarantees that all of BLASTP computational jobs on mappers can be completed, even if some of the mappers stop.

### 2.1. GPU Programming

As the GPU has become increasingly more powerful and ubiquitous, researchers have begun developing various nongraphics or general-purpose applications [18]. Traditionally, the GPUs are organized in a streaming, data-parallel model in which the coprocessors execute the same instructions on multiple data streams simultaneously. Modern GPUs include several (tens to hundreds) of each type of stream processor; both of graphical and general-purpose applications thus are faced with parallelization challenges [19].

Nvidia released the compute unified device architecture (CUDA) SDK to assist developers in creating nongraphics applications that run on GPUs. CUDA programs typically consist of a component that runs on the CPU, or host, and a smaller but computationally intensive component called the kernel that runs in parallel on the GPU. Input data for the kernel must be copied to the GPU's on-board memory from CPU's main memory through the PCI-E bus prior to invoking the kernel, and output data also should be written to the GPU's memory first. All memory used by the kernel should be preallocated.

**Figure 1:**
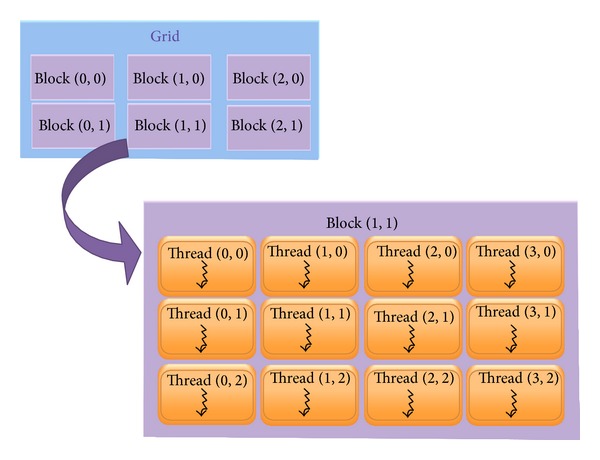
The parallelism architecture of GPGPU.

Kernel executes a collection of threads that computes a result for a small segment of data. To manage multiple threads, kernel is partitioned into thread blocks, with each thread block being limited to a maximum of 512 threads. The thread blocks are usually positioned within a one- or two-dimensional grid. Each thread can be positioned within a given block where it belongs, and this given block can be positioned within the grid. Therefore, each thread can calculate which elements of data to operate on and which regions of memory to writhe output to by an algebraic formula. Each block is executed by a single multiprocessor, which allows all threads within the block to communicate through on-chip shared memory. The parallelism architecture of GPGPU is illustrated in Figure 1.

### 2.2. Hadoop Framework

**Figure 2:**
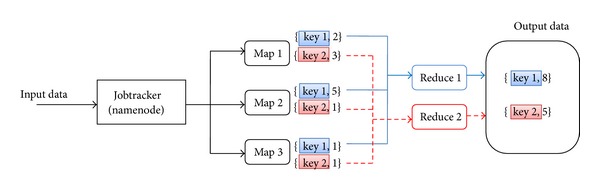
Computation of Map/Reduce framework of Hadoop.

Hadoop is a software framework to copy with distributed data in parallel by communicating computing nodes. Hadoop runs data-intensive applications through the Map/Reduce parallel processing technique. This framework has been used in many cloud industry companies, such as Yahoo, Amazon EC2, IBM, and Google. The example of computation of Map/Reduce framework is illustrated in Figure 2. In the mapper stage, the input data is split into smaller chunks corresponding to the number of mappers, and each mapper performs the operation on the data chuck. Output of each mapper has the format of 〈key, value〉 pairs. Outputs from all mappers, 〈key, value〉 pairs, are classified by key before being distributed to reducer. Reducer adds values by the same key. Outputs of reducers are 〈key, value〉 pairs where each key is unique.

**Figure 3:**
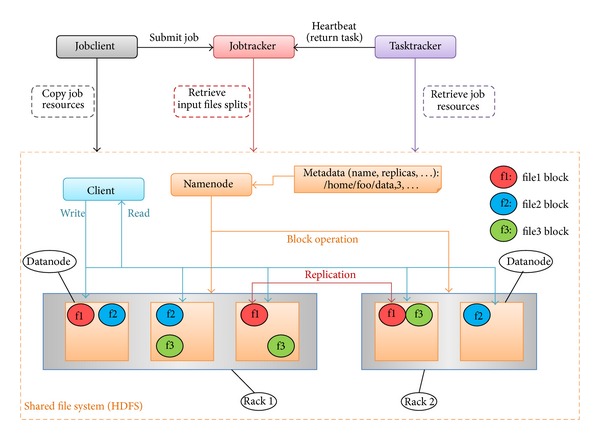
The architecture of Hadoop cluster and HDFS.

Hadoop cluster consists of a single master and multiple slave nodes. The role of the master node is a jobtracker, tasktracker, namenode, and datanode. A slave node, as computing node, is a datanode and tasktracker. The jobtracker is the service within Hadoop that manages Map/Reduce tasks that can be completed on computing nodes in the cluster, the nodes that already have the data. A tasktracker is a node in the cluster that accepts tasks and maps, reduces, and shuffles operations from a jobtracker. The architecture of Hadoop cluster is shown in Figure 3.

Hadoop distributed file system (HDFS) is the distribution file system used by Hadoop framework in default. Each input data file is split into data blocks that are distributed on datanodes by HDFS. HDFS can create multiple replicas of data blocks and distributes them on datanodes usually in the same rack as the source datanode throughout a cluster to enable reliable, extremely rapid computations. The namenode serves as both a directory namespace manager and a node metadata manager for the HDFS. There is a single namenode running in the HDFS architecture. The architecture of HDFS is shown in Figure 3.

### 2.3. BLASTP

The basic local alignment search tool (BLAST) [20], as it is commonly referred to, is a database search tool, developed and maintained by the National Center for Biotechnology Information (NCBI). The web-based tool for BLAST searches is available at http://blast.ncbi.nlm.nih.gov/Blast.cgi.

The BLAST suite of programs has been designed to find high scoring local alignments between sequences, without compromising the speed of such searches. BLAST uses a heuristic algorithm which seeks local as opposed to global alignments and is therefore able to detect relationships among sequences which share only isolated regions of similarity [20]. The first version of BLAST was released in 1990 and allowed users to perform ungapped searches only. The second version of BLAST, released in 1997, allowed gapped searches [21]. BLASTP is used for both identifying a query amino acid sequence and finding similar sequences in protein databases. BLASTP has three implementations on GPU, GPU-NCBI-BLAST, CUDA-BLASTP, and GPU-BLASTP. All three implementations achieve 4x~40x speedup over a single-thread CPU implementation of NCBI-BLAST.

### 2.4. Cloud-BLASTP

**Figure 4:**
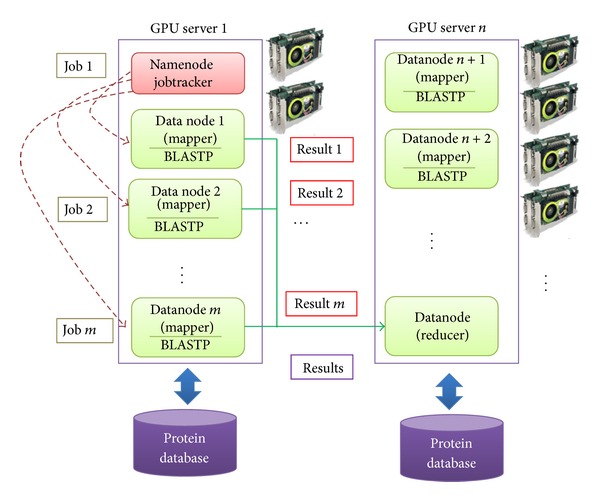
The architecture of Cloud-BLASTP.

To enhance the performance of CUDA-BLASTP on single GPU is to scale with multiple GPUs. In the proposed distributed GPU system, we utilized Hadoop framework to manage multiple GPUs. The Cloud-BLASTP architecture is demonstrated in Figure 4. Each single GPU server has a GPU card. To derive these distributed GPU cards, Hadoop is suitable for managing these cards. Every mapper in a node performs BLASTP and a reducer collects all the results produced by mappers. In this architecture, the sequence database is separated into several parts and uploaded to servers by HDFS. The features of Hadoop BLASTP are high performance, availability and reliability, and scalability.

#### 2.4.1. High Performance

In Hadoop BLASTP, the BLASTP jobs are performed in parallel by Map/Reduce framework. The number of the BLASTP jobs can be performed simultaneously which is the same as the number of the mappers. If the number of the BLASTP jobs is greater than the number of the mappers, then the number of mappers will assign the rest of unperformed BLASTP jobs to available mappers immediately.

#### 2.4.2. Availability and Reliability

Hadoop BLASTP is able to avoid the BLASTP jobs stop when mappers are down. By using Hadoop fault tolerance mechanism, when a datanode (mapper) is down during BLASTP computation, its BLASTP job will be reassigned to another slave node (mapper) by namenode. Therefore, all of the submitted BLASTP jobs never stop because one of the datanodes fails in Hadoop BLASTP. A hardware failure on the physical server causes a disastrous failure as all mappers running on it die. One way is that all of these jobs can be reassigned, and another way is that several new mappers are created on available hosts and then these jobs are reassigned to these new mappers. Thus, Hadoop BLASTP has high availability.

#### 2.4.3. Scalability

By Hadoop framework, Hadoop BLASTP can create new slave mappers as datanodes according to the number of submitted BLASTP jobs. When large amounts of the BLASTP jobs are submitted, Hadoop BLASTP can create more mappers to copy with more BLASTP jobs to enhance the performance.

## 3. Cloud-BLASTP Platform

Cloud-BLASTP is a protein alignment cloud service under Hadoop framework, BLASTP, and GPU architecture. The cloud computing platform is composed of one NFS server and 4 GPU servers in the Providence University Cloud Computation Laboratory. Each server is equipped with an Intel i7 3930 3.2 GHz CPU, 16 G RAM, and Nvidia GeForceGTS 480 graphics card (Fermi architecture). Each server is running under the O.S. Ubuntu version 10.4 with Hadoop version 0.2 Map/Reduce framework. Each server is responsible for a map operation and a reduce operation. The total number of the Map/Reduce operations is up to 4, respectively.

## 4. Performance Evaluation

**Figure 5:**
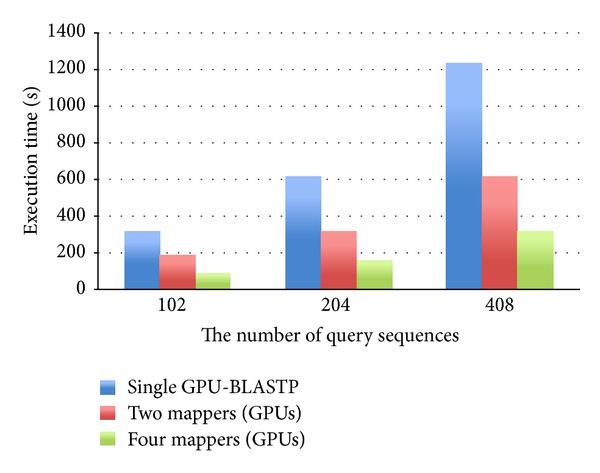
The performance of Cloud-BLASTP in the various numbers of mappers.

To assess the performance of the proposed cloud service, we compared the execution time between stand-alone BLASTP and Cloud-BLASTP. The performance of both programs depends upon the amount of data set and the number of computing mappers. Therefore, the performance between the programs is tested with respect to these two factors. In the first experiment, the data size of protein database is 841 MB, and the numbers of query sequences are 102, 204, and 408. The number of query sequences processed by each mapper is the number of query sequences divided by the number of mappers. For example, suppose there are two mappers, and mapper 1 has to process 26 sequences and mapper 2 has to process 25 sequences. The results are shown in Figure 5. As shown in the figure, the execution time of comparing 102 sequences can be reduced from 318 seconds (consumed by the single GPU-BLASTP) to 187 seconds and 88 seconds by executing Cloud-BLASTP with 2 and 4 mappers, respectively. Also, the execution time of comparing 204 sequences can be reduced from 622 seconds to 318 seconds and 164 seconds by executing Cloud-BLASTP with 2 and 4 mappers, respectively. For querying 408 sequences, the execution time can be reduced from 1236 seconds to 622 seconds and 318 sequences by executing Cloud-BLASTP with 2 and 4 mappers, respectively. It is obvious that with less mappers (GPU servers) the performance is much worse. Clearly, the execution time is effectively reduced when more mappers are involved. In general, more mappers achieve a faster processing speed.

**Figure 6:**
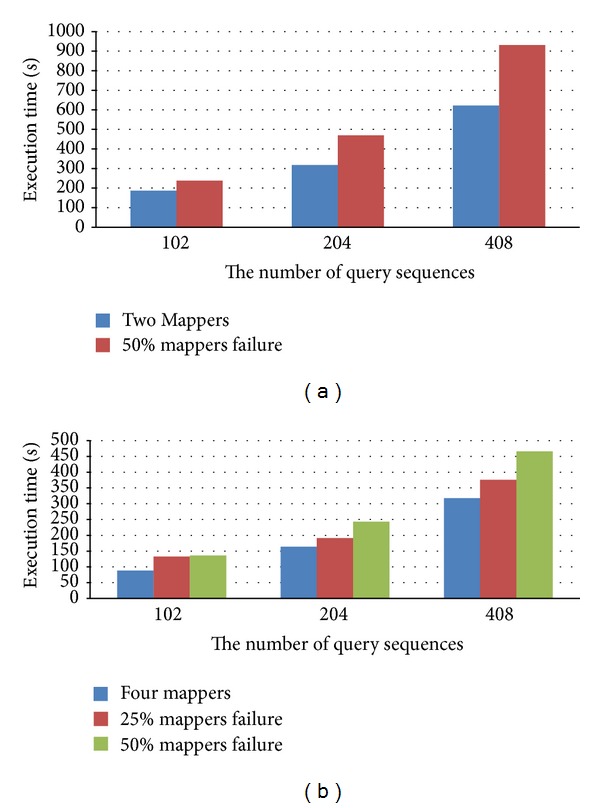
Execution time of node failure at half of execution duration of Cloud-BLASTP. (a) Two mappers; (b) four mappers.

**Figure 7:**
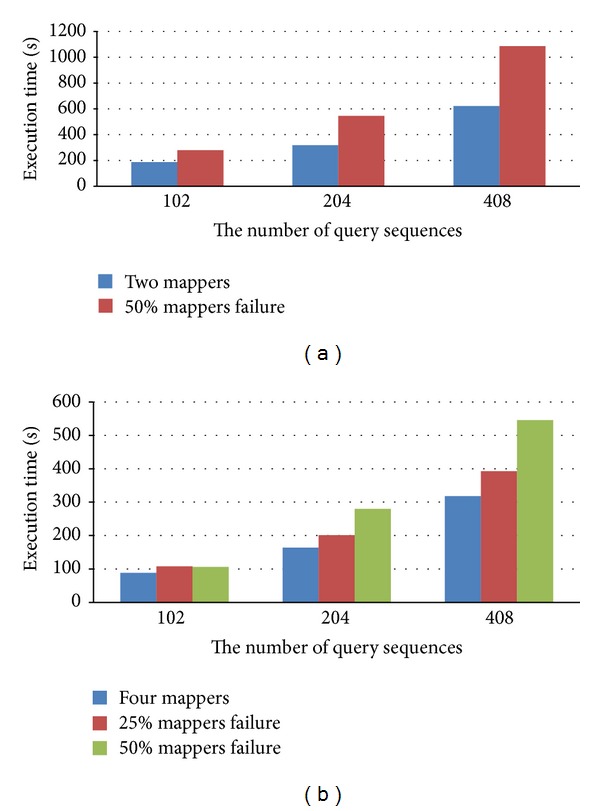
Execution time of node failure at 25% of execution duration of Cloud-BLASTP. (a) Two mappers; (b) four mappers.

In Cloud-BLASTP, the important features are reliability and availability. The computing process at the failed node is able to continue at another node that has the replica of data of the failed node. Therefore, we performed a simulation to evaluate the reliability and availability of the proposed cloud service when mappers fail. In this simulation, we make half of the mappers fail in the duration of executing BLASTP. In this simulation, the heartbeat time is set to one minute, and the number of replicas is set to three as default. Therefore, all of jobs can be completed even when some of the nodes fail. Figures 6(a) and 6(b) demonstrate the performance of the proposed method meeting corresponding half of mappers fail and quarter of mappers fail for querying 102, 204 and 408 sequences when failures happen at duration of 50% execution, respectively. The execution time with no failure is shown as the blue bar, and the execution time with failure in a half of mappers is shown as red bar. From the experiment results, it shows that the jobs can be completed when mappers fail, but the execution time is more than normal execution time because the failed jobs have to be assigned to other health mappers. Figures 7(a) and 7(b) demonstrate the performance when the failures happen at the duration of 25% execution. Although the mappers fail, the execution time of redundancy is related to the number of mappers too. Thereby, Cloud-BLASTP is mapper failure-free.

## 5. Conclusion

In the past few years, sequencing technologies have grown rapidly. The amount of produced sequence data is from gigabase increased to terabase, and the duration is from months decreased to days. Therefore, the performance of the bioinformatics tools is important to analyze data efficiently. Sequence alignment is the basic and common analysis step for biologists to practice further experiment. BLASTP is one of the wild-used local alignment tools for protein sequences. It is now provided on NCBI organization. BLASTP has also been implemented on GPU to enhance the alignment performance. Although BLASTP outperforms most existing local sequence alignment tools, it does not satisfy the need of high scalability and high availability for searching huge protein database.

Hadoop framework has become popular for providing efficient and available distributed computation to users. In this paper, we propose a cloud computing tool, called Cloud-BLASTP, for protein local alignment by integrating Hadoop framework and BLASTP tool. Cloud-BLASTP takes advantage of high performance, availability, reliability, and scalability. Cloud-BLASTP guarantees that all submitted jobs are properly completed, even when running job on an individual node or mapper experience failure. The performance experiment shows that it is desirable for biologists to investigate the protein structure and function analysis by comparing large protein database under reasonable time constraints.
